# Regulation of Malignant Myeloid Leukemia by Mesenchymal Stem Cells

**DOI:** 10.3389/fcell.2022.857045

**Published:** 2022-06-08

**Authors:** Zhenya Tan, Chen Kan, Mandy Wong, Minqiong Sun, Yakun Liu, Fan Yang, Siying Wang, Hong Zheng

**Affiliations:** ^1^ Department of Pathophysiology, Anhui Medical University, Hefei, China; ^2^ Department of Biological Sciences, Georgia Institute of Technology, Atlanta, GA, United States

**Keywords:** mesenchymal stromal cells, leukemic stem cells, bone marrow microenvironment, hematopoietic stem cell niche, leukemic progression

## Abstract

Bone marrow microenvironment (BMM) has been proven to have benefits for both normal hematopoietic stem cell niche and pathological leukemic stem cell niche. In fact, the pathological leukemia microenvironment reprograms bone marrow niche cells, especially mesenchymal stem cells for leukemia progression, chemoresistance and relapse. The growth and differentiation of MSCs are modulated by leukemia stem cells. Moreover, chromatin abnormality of mesenchymal stem cells is sufficient for leukemia initiation. Here, we summarize the detailed relationship between MSC and leukemia. MSCs can actively and passively regulate the progression of myelogenous leukemia through cell-to-cell contact, cytokine-receptor interaction, and exosome communication. These behaviors benefit LSCs proliferation and survival and inhibit physiological hematopoiesis. Finally, we describe the recent advances in therapy targeting MSC hoping to provide new perspectives and therapeutic strategies for leukemia.

## Introduction

HSC niche, including support cells and support cytokines, takes part in the process of HSC generation, self-renewal, proliferation, and differentiation (Morrison and Scadden, 2014). The external or self-microenvironment dynamically changes the niche components, leading to proliferation or differentiation of HSCs, which result in the controllable generation of leukocytes or erythrocytes for maintaining the internal biological homeostasis (Kumar and Geiger, 2017). Physiologically, long-term hematopoietic stem cells exist in the stable endosteum microenvironment. The microenvironment maintains a low-oxygen environment, and it is sustained by the physical interactions and various cytokines that derived from support cells such as mesenchymal stem cells, endothelial cells, and megakaryocytes. Then, the short-term hematopoietic stem cells mobilize the perivascular microenvironment for further activation, resulting in the loss of homeostasis and the generation of hematopoietic progenitor cells (Pinho and Frenette, 2019). All actions of HSCs are strictly regulated by their physiological requirements. However, in some pathological conditions, uncontrollable HSC niche and HSC changes mobilize hyperactivity of HSCs to differentiate to plethoric leukocytes or erythrocytes, causing ineffective hematopoiesis for leukemia initiation (Shlush and Feldman, 2021). With the advent of chemotherapy and immunotherapy, the 5-year survival rate of leukemia has shown a significant increase. However, some patients still show drug insensitivity or chemoresistance, which is the major barrier for complete leukemia cure. In fact, from the perspective of the bone marrow microenvironment, it is difficult to completely cure leukemia because of the protection of BMM. Therefore, disintegrating the leukemic stem cell (LSC)-hiding bone marrow microenvironment can be used as a new therapeutic strategy.

The endosteal microenvironment of HSCs has been widely accepted by the public and lots of hematopoiesis-related cellular and molecular components have been recently confirmed. Mesenchymal stem/progenitor cells, osteoblasts, endothelial cells, perivascular cells, megakaryocytes, immune cells, Schwann cells, and so on all mainly attract HSCs and maintain HSC self-renewal for hematopoietic homeostasis. Besides, in response to external stress or self-proliferation signaling, HSCs also remodel bone marrow microenvironment (BMM) for its proliferation and differentiation, mainly through directly cell-to-cell contact or cytokines secretion (Batsivari et al., 2020). However, pathologically, leukemic stem cells also have pathological LSC niche components similar to HSC niche. The LSC niche also maintains the stemness of LSCs and promotes LSC proliferation and escape from immune cells attack or drug targeting (Mendez-Ferrer et al., 2020). More importantly, increasing evidence proves heterogeneous BMM can influence or even control leukemogenesis. For example, miR-126 secreted by endothelial cells in BM supply quiescence and leukemia growth of BCR-ABL^+^ LSCs (Zhang et al., 2018) and activated β-catenin in osteoblasts was sufficient to lead to acute myeloid leukemia (Kode et al., 2014). Another recent article showed that blood bacteria-induced up-regulated IL-6 leads to pre-leukemic myeloid hyperproliferation in Tet2^−/−^ HSCs, however, merely Tet2 deficiency can hardly cause pre-leukemic phenotype (Meisel et al., 2018), which suggests that dysregulated non-hematopoietic cells and chromosomal mutative HSCs together instigate malignant leukemia.

Mesenchymal stem cells, as adult pluripotent stem cells, can ultimately differentiate into adipocytes, osteoblasts, and chondrocytes to regulate physical growth and tissue injury repair (Berger et al., 2016; Qu et al., 2016; Shu et al., 2018). Due to its characteristics of histocompatibility and multi-directional differentiation, MSC research has made great progress in the fields of regenerative medicine, autoimmune diseases, and immunoregulation, i.e., bone tissue regeneration and graft-versus-host disease inhibition (Singer and Caplan, 2011; Frenette et al., 2013). On the other hand, it can act as HSC niche cells to maintain hematopoietic homeostasis (Dong et al., 2016). Bone marrow MSCs are heterogeneous, and various MSC subtypes including Nestin^+^, Prx1^+^, SP7^+^, Leptin receptor-expressing and CXCL12-abundant reticular cells are involved in HSCs’ homeostasis (Morrison and Scadden, 2014). MSCs maintain and protect HSC self-renewal, proliferation, and differentiation. Different progeny of MSCs associates HSCs and, in general, most of them are known to secrete HSC-supporting factors, such as C-X-C motif chemokine ligand 12 (CXCL12), angiopoietin, stem cell factor (SCF/Kit ligand), and others (Asada et al., 2017). In fact, leukemic MSCs are also essential for leukemia progression. Accumulating evidence suggested that the altered BMM in general, and particularly in mesenchymal stem cells (MSCs) and their progeny, plays a pivotal role in the evolution and propagation of leukemia (Kode et al., 2014; Schepers et al., 2015). Heterogeneous BMM accelerates the leukemia progression with non-cell autonomous manner, coordinates chromosomal aberrations of leukemic cells. The crosstalk between LSCs and the associated BMM represents a powerful relationship that influences leukemia initiation, progression, and response to therapy (Hanoun and Frenette, 2013; Shlush et al., 2014; Zhou et al., 2016).

Currently, the role of tumor microenvironment in neoplasm initiation and malignant evolution has been increasingly recognized. However, the contribution of bone marrow mesenchymal stem cells to disease progression remains poorly explored. This review puts emphasis on our current knowledge of the involvement of LSCs and associated MSCs in processes facilitating leukemia pathogenesis and progression. Moreover, this review provides a hint of new therapeutic strategy targeting, targeting not only gene-mutant HSCs but also disordered BMM may rapidly and thoroughly cure different types of leukemia (Cao et al., 2020; Borella et al., 2021).

## LSCs Facilitate Transformation of MSCs Into LSC-Beneficial Niche

BMM plays an irreplaceable role in physiological hematopoietic stem cells niche and pathological leukemic stem cells niche. BM niche cells (i.e., MSCs) and HSCs interact to regulate its resting adhesion, proliferation, and differentiation. Mesenchymal stem cells as the most important HSC niche cells have been shown to principally maintain the stabilization of HSCs, meanwhile flexibly regulate its proliferation and differentiation (Mendez-Ferrer et al., 2008) through both direct cell-to-cell contact and cytokine-receptor interactions. The BMM of HSCs and LSCs have similar natures, since MSCs, osteoblasts, endothelial cells are essential for both healthy HSCs and malignant LSCs, and the spatial localization (Boyd et al., 2014) and molecular phenotype of LSCs have no obvious differences from those of HSCs. In leukemia, LSCs and HSCs form a competitive relationship in BMM with the dominant and minor clones. Obviously, LSCs are still leading advantages in proliferation, differentiation, and propagating, lots of changes in the transcriptions and chemokines make it easier to regulate the malignant transformation and impair hematopoiesis (Medyouf et al., 2014; Waclawiczek et al., 2020). Importantly, normal and leukemic MSCs all harbor LSCs pathologically, including promoting location, growth, expansion, and apoptosis inhibition to promote leukemic process (Brenner et al., 2017). Moreover, MSCs in leukemia show disorganized feature regardless of leukemic type. Avanzini et al. identified a gene aberrant mutation of MSCs in MPN is more frequent compared to healthy MSCs, and patients with genetically aberrant MSCs have higher myelopoiesis and spleen index (Avanzini et al., 2014). Hence, the role of MSCs in leukemia should be transformed from a supporter of LSCs into a promoter of leukemia alongside with LSCs. The genetic phenotype of MSCs is rarely detected in current clinical diagnosis, and the combination of MSCs and HSCs gene sequencing will be more conducive to the diagnosis and prognosis of leukemia.

**FIGURE 1:**
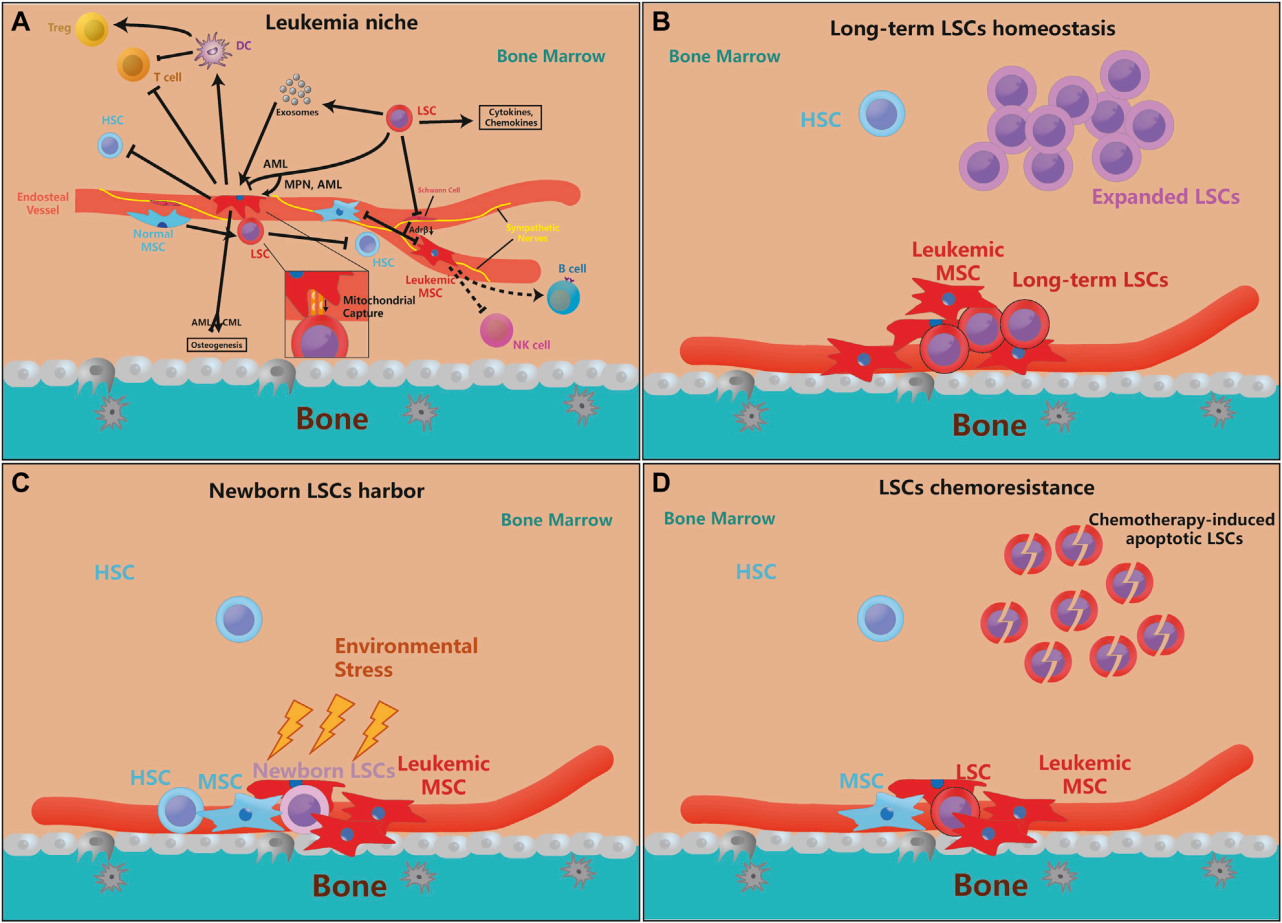
MSCs are essential for leukemia initiation and progression. **(A)**. The pathological LSCs drive MSCs to leukemic-permissible MSCs through physical interactions, cytokines, chemokines, and exosomal secretion, and regulation of their osteogenesis ability. Moreover, leukemic MSCs inhibit the stemness and self-renewal of normal HSCs, but physiological MSCs also promote the proliferation and anti-apoptosis ability of LSCs for their benefits. On the other hand, LSCs can also denervate SNS and inhibit the release of Adrβ, making their inhibition of MSCs ineffective. Furthermore, leukemic MSC can directly or indirectly inhibit T cell activity and proliferate Tregs, but its function on B cells, etc. is not yet clear. Besides, more evidence reveals that genetically mutated MSCs are enough to motivate HSCs leukemogenesis (the arrow means that the source cell promotes the proliferation or function of target cell, and the inhibitory symbol means that the source cell inhibits the proliferation or function of target cell). **(B–D)**. The supporting effect of MSCs on LSCs is mainly in three aspects, which is the maintenance of quiescent LSCs, the protection of newborn LSCs, and LSCs chemoresistance. Some newly mutated LSCs need the protection of MSCs to avoid environmental stress and evade immune surveillance. MSCs can also protect a part of quiescent LSCs to maintain the ability for long-term leukemogenesis, while the expansion of LSCs does not depend on MSCs very much. When chemotherapy kills LSCs, MSCs can help LSCs chemoresistance and promote recurrence.

It is necessary to clarify a more specific leukemia microenvironment, how MSCs give LSCs advantages and how LSCs actively change the MSC phenotypes are unclear now. Next, we will elaborate on this process from the perspective of both LSCs and MSCs (Figure 1). From the perspective of LSCs, chromatin aberrations in LSCs autonomously adjust the primordial BMM to adapt its leukemogenic effects. Moreover, LSCs actively alter the properties of MSCs, which always makes MSCs beneficial for LSC survival and proliferation, but the reprogramming of MSCs in different leukemia subtypes is still variant. For example, CML LT-HSCs (as well as LSCs) secrete MIP-1, IL-6, and G-CSF to decrease the secretion of CXCL12, which is the essential chemokine for HSC maintenance (Greenbaum et al., 2013), in MSCs for its own competitive advantage (Zhang et al., 2012; Agarwal et al., 2019), whereas CML LSCs hardly need CXCL12 for its expansion. However, in AML bone marrow context, AML-derived MSCs express no significant change in CXCL12 (Geyh et al., 2016), but CXCR4, the receptor of CXCL12, is overactivated by AML cells (Zeng et al., 2009). Hence LSCs-influenced MSCs could be different depending on the context of LSCs, chronic, acute myeloid leukemia or MDS LSCs remodel BMM into respective cultivating-conducive environment. More importantly, in addition to the differences in chemokine secretion between the two types of MSCs, the capacity of MSCs differentiation is also changed in leukemia. It is known that CML patients often develop excessive trabecular bone and bone thickness, whereas patients with AML do the opposite (Krause et al., 2013). Similarly, MSCs are also dynamically changed in different leukemic subtypes. Schepers et al. found TPO and CCL3, in conjunction with direct interactions between MSCs and BCR-ABL + leukemic myeloid cells, derive the overproduction of osteoblast derivatives and myelofibrosis during MPN development (Schepers et al., 2013), but bone marrow MSCs from pre-AML MDS and AML patients display apoptosis, deficient proliferation rate, and impairment of osteogenic differentiation (Geyh et al., 2013; Geyh et al., 2016; Li et al., 2020), increased adipogenic potential with improved ability to support survival of leukemia progenitor cells (Azadniv et al., 2020). Moreover, although progressive AML LSCs hardly need the support from MSCs for its expansion, early weak AML LSCs engraftment still need MSCs for its survival and niche reconstruction (Xiao et al., 2018). However, recently it was reported that MSCs are proliferative and over-differentiated into pre-osteoblasts and osteogenic progenitor cells in the AML transplant mouse model (Hanoun et al., 2014). Based on recent research that BMM is spatiotemporally different with AML infiltration (Duarte et al., 2018), it can be considered that MSCs in AML are dynamically changing with AML infiltrated progression. However, the dynamic regulation process of CML by MSCs has not been reported yet.

On the other hand, large-scale whole exome sequencing had not found obvious mutated MSCs in leukemia patients (von der Heide et al., 2017), so leukemic MSCs are partly derived from epigenetic modifications of normal MSCs in AML and MDS patients. Those leukemic MSCs are aging, growth deficiency and osteogenic differentiation (Geyh et al., 2013; Geyh et al., 2016). The function-related gene sets, such as TBX15, PITX2, HOXB6, are regulated by relevant specific hypermethylation signals (Geyh et al., 2016; Bandara et al., 2021). At the same time, multiple studies have confirmed that multiple methylases strictly control the cell stemness, senescence (Cakouros and Gronthos, 2020) and differentiation functions (Ye et al., 2012) of MSCs (Sui et al., 2020), and the loss of KDM4B mimics a leukemia-like MSCs(Deng et al., 2021), suggesting that leukemia cells epigenetic change MSCs for its BMM remodeling. Applicably, CM-272, the inhibitor of DNMTs and G9a, alleviates multiple myeloma (MM) and bone loss by removing hypermethylation of osteogenic regulators (Garcia-Gomez et al., 2021). Another hypomethylating drug azacitidine targets hypermethylated MSCs for MDS remission (Poon et al., 2019), and reversely supports healthy hematopoiesis (Wenk et al., 2018). So specific demethylating medicines can restore the growth and osteogenic differentiation of MSCs and are potentially effectivity for the treatment of AML and MDS. At the same time, low-expressed METTL3 modulates chemoresistance in AML by promoting the adipogenic differentiation of MSCs, which indicating chemo-sensitization of epigenetic modification (Pan et al., 2021).

Functionally, single-cell transcriptome demonstrated that LEPR + mesenchymal stem cells are the most important leukemic support cell that is dysfunction in AML, and a series of niche factors that induce down-regulation by AML were identified, such as CXCL12, KITL, ANGPT1, and VCAM1 (Baryawno et al., 2019). Another AML-preserved NESTIN + MSCs subcluster was reported to enhance leukemic blast bioenergetics by increasing OXPHOS and TCA cycle, and antioxidant defenses for facilitating chemoresistance (Forte et al., 2020). More amazingly, malignant hematopoietic cells are more active in changing their niche than imagined. In contrast to the classical Warburg effect, AML cells capture mitochondria from super-oxidized MSCs by leukemia-derived tunneling nanotubes to produce excess ATP, increase regrowing potential and get a better survival (Moschoi et al., 2016; Marlein et al., 2017). This mitochondrial transfer function is important for AML cells to respond to killing resistance, oxidative stress restriction, cellular respiratory function, and healthy mitochondrial mass maintaining (Burt et al., 2019; Saito et al., 2021) and can be terminated by CD38 antibody (Mistry et al., 2021), but T-ALL cells reversely transfer its damaged mitochondria into MSCs for ROS elimination and chemoresistance (Wang et al., 2018). Interestingly, the MSCs in leukemia are functionally distinct from normal MSCs, the confusion of leukemic MSCs could have cell-autonomous and non-cell-autonomous detrimental effects to adjacent healthy HSCs. Leukemia-related HSCs show insufficient hematopoiesis, reduce homing, and impair growth when exposed to leukemic MSCs (Geyh et al., 2013; Geyh et al., 2016; Mead et al., 2017). Therefore, restoring MSCs before HSC transplantation could promote the therapy effect and reduce relapse.

## Karyotype Aberrations in MSCs Are Sufficient to Induce Leukemia

From the respective of LSCs niche, because leukemia patients who accompanied by karyotype aberrations in MSCs often show a worse prognosis (Blau et al., 2011), accumulated evidences prove that changes in non-hematopoietic niche cells, rather than HSCs, can adequately induce leukemia. At first, as is shown by Rupec et al., IκBα-deficient fetal liver in mice is sufficient for MPN initiation (Rupec et al., 2005), further more evidence points that HSC niche cells, especially MSCs, are also the pathogenesis of leukemia (Dong et al., 2016). Walkley et al. found that null RAR-γ or Rb developed myeloproliferative syndromes due to the deficiency of RAR-γ or Rb in bone marrow microenvironment (Walkley et al., 2007a; Walkley et al., 2007b). Deficient Dicer1, an essential enzyme for microRNA biogenesis in MSCs and osteoprogenitors, was reported to impair osteogenesis and cause mitochondrial damage and genotoxic stress in HSCs which could finally evolve to leukemia (Raaijmakers et al., 2010; Zambetti et al., 2016). We notice that these genes are related to cell stemness and homeostasis, which prove that the maintenance of MSCs self-renewal is essential for HSCs homeostasis. The long-term loss of MSCs homeostasis leads to HSCs instability and increased leukemia tendency; therefore, abnormal leukocyte proliferative diseases caused by changes in MSCs should receive more attention.

On the other hand, long-term inflammatory stimuli derived from MSCs can also drive HSCs loss-of-homeostasis, excessive myelopoiesis, and leukemia. Notch deficiency in BM MSCs and endothelial cells activate the miR155/NF-κB/G-CSF/TNFα inflammation pathway to develop a myeloproliferative disease symptom such as hepatosplenomegaly, anemia, and granulocytosis (Kim et al., 2008; Wang et al., 2014); meanwhile, leukemogenic effects of fibrosis-related MSCs not only decrease hematopoietic support but also secrete pro-inflammatory s100a8/9 for HSCs-exhausted MPN and subsequent MDS initiation (Leimkuhler et al., 2021). PTPN11 activating mutations were always found in Noonan syndrome patients, and it is known that PTPN11 activating mutation always causes leukemia, such as JMML, AML, or ALL. In the past, it was always thought that this was the result of PTPN11 activating mutations in HSCs, but we recently found that activating mutation of PTPN11 in MSCs, not HSCs, activates HSCs through long-term pro-inflammatory signals from MSCs and monocytes, which leads to excessive activation and proliferation of HSCs and the eventual progression to MPN (Dong et al., 2016). Another type of CD90^−^CD13^−^CD44 ^+^ proangiogenic mesenchymal cancer stem cell was considered to have potential tumorigenic ability and AML support ability, but whether there was the presence of chromosomal aberration had not been explored (Cao et al., 2020). Those above illustrate a new model of leukemia that MSCs induce a sufficient inflammatory environment that stimulates the initiation of leukemia (Table 1).

**TABLE 1:** Multiple mutations in MSCs induce leukemogenesis.

Mice Model	Mutant gene	Labeling strategy	Phenotype	References
pRb^fl/fl^ Mx1-cre	Rb	Mesenchymal cells	MPN	Walkley et al. (2007b)
RARγ^−/-^	RARγ	BMM	MPN	Walkley et al. (2007a)
Mib^fl/fl^ MMTV-cre	Notch	Mesenchymal cells	MPN	Kim et al. (2008); Wang et al. (2014)
Mib^fl/fl^ Mx1-cre				
RBPJ^fl/fl^ Mx1-cre				
Dicer^fl/fl^ Osx-GFP-Cre	Dicer Sbds	osteoprogenitors	MDS	Raaijmakers et al. (2010); Zambetti et al. (2016)
Sbds^fl/fl^ Osx-GFP-Cre				
PTPN11^fl/fl^ Nestin-cre	PTPN11	Mesenchymal cells	MPN	Dong et al. (2016)

## Abnormalities in Cytokines, Chemokines and Signaling Pathways Occur in Both LSCs and MSCs

Firstly, the complex cytokines network is the main messenger of communication between MSCs and LSCs. The binding of these cytokines to their receptors mediates downstream signaling cascades, which in turn lead to changes in cell behavior (Geyh et al., 2013; Brenner et al., 2017). Table 2 details the categorization of functionally similar cytokines, summarizes these cytokines and important signaling proteins, and aims to sort out a detailed communication network. The functions of these cytokines are mainly related to two major categories: the LSCs support and HSCs impairing, and the inflammatory environment and bone remodeling. It is worth mentioning that due to mutual crosstalk, their functions are variable. For example, MSCs-derived N-cadherin not only impels LSCs adhesion and expansion, but also protects LSCs from TKI (Zhang et al., 2013; Medyouf et al., 2014). Meanwhile, a number of inflammation-associated cytokines such as CCL3, TNF, IL-6, IL-8, and the like have been identified to be widely expressed in the crosstalk of MSCs and leukemic cells, and it has been previously shown that the inflammatory BMM is indispensable in leukemia formation (Meisel et al., 2018). Therefore, inhibiting the inflammatory response in the BMM may cut off the association between LSCs and BMM and may increase the sensitivity of TKI.

**TABLE 2:** Numerous cytokines and chemokines are involved in leukemia niche support.

Effector	Origin	Species	Effect	References
LSC supporting and normal HSC impairing				
Jagged1	MSC overexpress	AML patients	Leukemic cells support	Geyh et al. (2016)
CXCL12, SCF, IGF-1	MSC low-express	AML engrafted murine model	Residual HSC mobilization	Huan et al. (2015); Kumar et al. (2018)
STC1, PDK1, GLUT1	MSC overexpress	AML patients	impairing hematopoiesis	Waclawiczek et al. (2020)
SCF, Angiopoietin-1	MSC low-express	MDS patients	Insufficient hematopoietic support	Geyh et al. (2013)
Jagged1, Osteopontin	MSC overexpress	MDS patients	Insufficient hematopoietic support	Geyh et al. (2013)
N-cadherin	MSC overexpress	CML patients	Cell adhesion and protecting CML cells	Zhang et al. (2013)
N-cadherin, IGFBP2,VEGFA,LIF	MSC overexpress	MDS patients	Enhance LSC expansion	Medyouf et al. (2014)
Inflammatory environment and endosteal remodeling				
CXCL2,TNF	LSC overexpress	MLL-AF9-driven murine model	Pro-inflammatory and anti-angiogenesis	Duarte et al. (2018)
TGF-β	LSC overexpress	MDS and AML patients	Compromising their immunomodulatory capability	Geyh et al. (2018)
NFKBIA	MSC overexpress	MDS patients	Inflammation attenuates hematopoiesis	Ping et al. (2019)
VCAM1	MSC overexpress	AML patients	VCAM1-VLA4 increase inflammatory factors and protect leukemic cells	Schmidt et al. (2011); Jacamo et al. (2014)
CCL3	MSC overexpress	Ptpn11-activating mutation murine model	Mediating leukemogenic effect	Dong et al. (2016)
IL-8, MMP9	MSC overexpress	CML patients and cell lines	Promoting CML progression and invasiveness	Corrado et al. (2016)
G-CSF, IL-6,MIP-1β	Leukemic cell overexpress	BCR-ABL-driven murine model	Decreasing CXCL12, support leukemic cell engraftment	Zhang et al. (2012)
CCL3,TPO	Leukemic cells overexpress	BCR-ABL-driven murine model	Endosteal osteoblasts expansion	Schepers et al. (2013)
IκBα, TNFα, CXCL1	MSC overexpress	BCR-ABL-driven murine model	Increasing inflammation and LSCs expansion	Schepers et al. (2013); Agarwal et al. (2021)
IL-6, TGF-β, TNFα	MSC overexpress	CML cell lines	Increasing MSC stress	Jafarzadeh et al. (2018)

**FIGURE 2:**
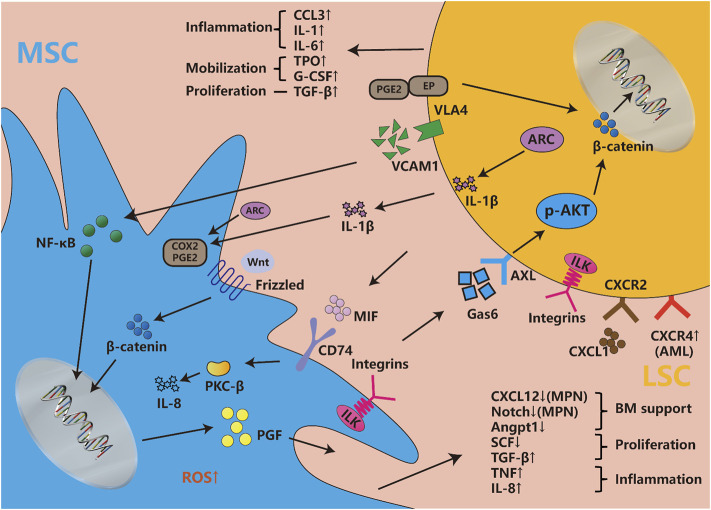
Cytokines, chemokines and signaling pathways influence both LSCs and MSCs. Abundant cytokines and chemokines are released by both LSCs and leukemic MSCs, which are mainly involved with the changes in inflammatory factors, proliferation and differentiation-associated cytokines, hematopoietic homeostasis-related chemokines, etc. They involve changes in multiple signaling pathways, such as the newfound VCAM1-VLA4-PGF/NF-κB, Gas6-AXL/p-AKT/β-catenin, MIF-PKCβ/IL-8, etc. Meanwhile, WNT/beta-catenin, Notch/Jagged, and other pathways are also disordered in leukemic niche. The integrin, considered to be widely expressed in both LSCs and MSCs, is involved in a series of classical pathways. The classic signaling pathway involved in cell proliferation and stemness such as MEK/ERK, JAK/STAST3, PI3K/AKT, etc., are not described in the figure, but they induce many changes in both types of cells.

Besides, as mentioned before that LSCs change leukemic MSCs adhesion, proliferation, differentiation, senescence, and epigenetics mainly through IL-6, CXCL12, TNF-α, angiopoietin, G-CSF, and so on. Recently, BMPs, an essential group of cytokines for osteogenesis, are also dysregulated in BMM, but the systematic role of BMPs in regulating BMM and leukemia has not been clearly explored (Doron et al., 2018; Zylbersztejn et al., 2018). Lots of signaling pathways participate in MSCs’ transformation as an executor of cellular function. Some signaling pathways like NOTCH, WNT, and TGF-β are essential for MSC physiological function, but LSCs could also change those signaling pathways to remodel MSC function into pathophysiological LSCs-supportive conditions. Both co-cultured leukemia cells and MSCs-activated PI3K/ILK/AKT, JAK/STAT3, MEK/ERK, and Notch/Hes signaling pathways support reciprocal survival and anti-apoptosis effects (Tabe et al., 2007). Moreover, the inflammatory signals released by LSCs activate MSCs and then maintain LSCs stemness and proliferation through feedback loops. LSCs were found to influence MSCs through MIF-PKCβ/IL-8 and VCAM1-VLA4- NF-κB/PIGF to program pro-inflammatory MSCs and hijack them for their own benefit (Schmidt et al., 2011; Abdul-Aziz et al., 2017). In response to the inflammation and angiogenesis environment of LSCs, Gas6 secreted from MSCs activates AXL/p-AKT/β-catenin to increase the self-renewal capacity of LSCs (Jin et al., 2017). Recent article found IL1β/COX2/PGE2/β-catenin/ARC reciprocal secretory loops promote the stability of the leukemia microenvironment and chemoresistance (Carter et al., 2019). Summarily, the crosstalk between MSCs and LSCs is complex multistage reactions. The cytokines and chemokines secreted by MSCs and LSCs directly interfere with their intracellular signaling pathways, then both cells regulate their model of proliferation and differentiation, and finally promote inflammatory factors release and angiogenesis to ensure the homeostasis and evolution of leukemia **(** Figure 2 **)**.

Interestingly, resistance and relapse caused by MSCs-protected LSCs in TKI have been widely recognized. Recent report found CXCR4 up-regulation by imatinib induces chronic myelogenous leukemia cell migration to bone marrow stroma and promotes survival of quiescent CML cells (Jin et al., 2008), while MSCs-derived Gas6 and N-cadherin stabilize or increase β-catenin levels in CML stem cells with or without TKIs and enhance maintenance of CML LSCs with anti-apoptosis and repopulating capacity (Zhang et al., 2013; Jin et al., 2017); inhibition of WNT/β-catenin signaling of MSCs prevents the development of MDS (Stoddart et al., 2017). Besides, combined JAK1/2 and Bcl2 inhibitors are another method to dismantle the protection of MSCs to LSCs (Karjalainen et al., 2017).

## Exosomes Link LSCs and MSCs as Major Non-cell-to-cell Contact Way

**FIGURE 3:**
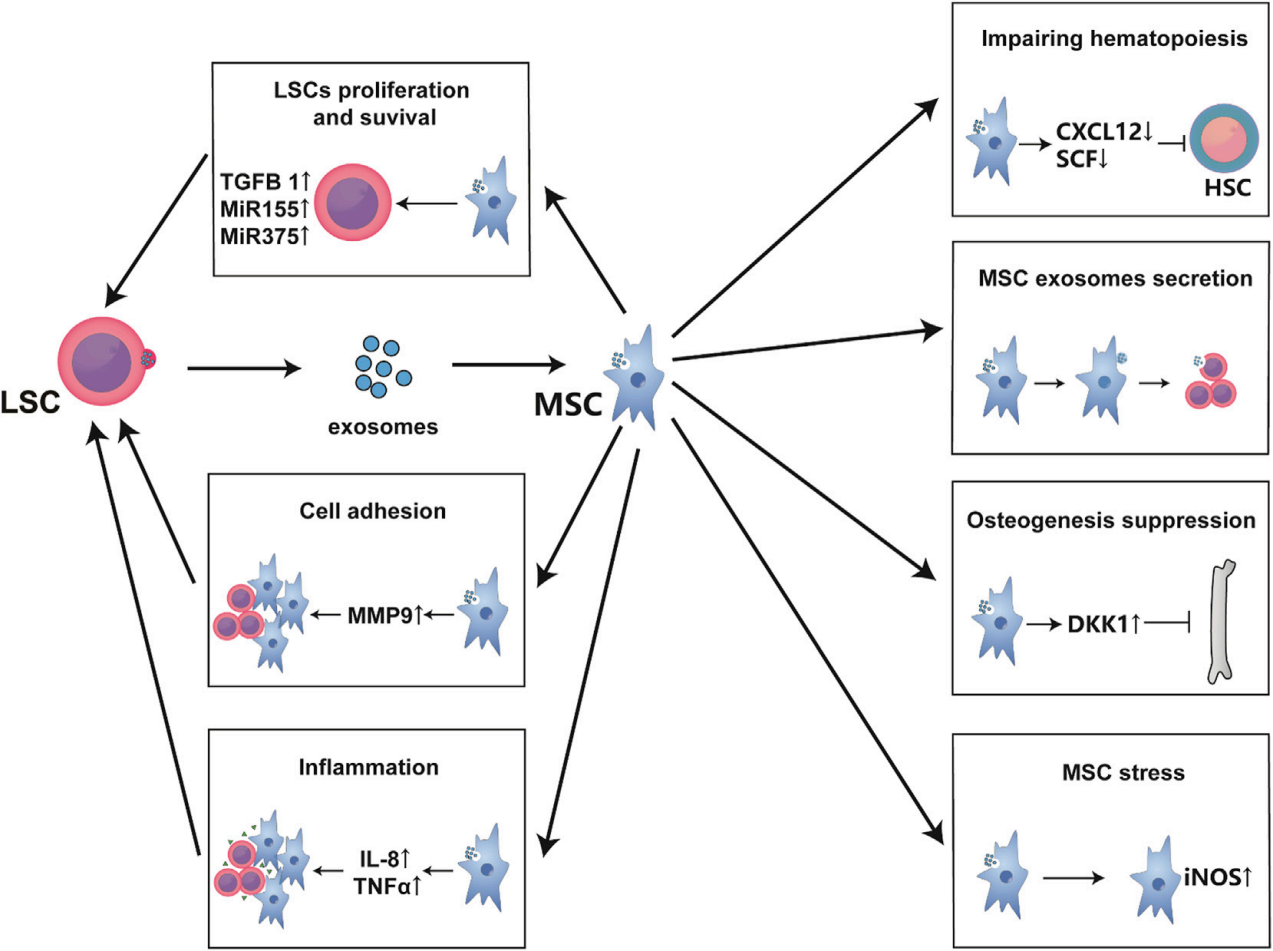
Exosomes are the main communication means between MSCs and LSCs. Exosomes secreted from LSCs are mainly absorbed by MSCs and endothelial cells. These exosomes usually alter the hematopoietic support function of MSCs to form a leukemia-permissive microenvironment mainly through the secretion of mass cytokines and microRNAs. As shown in the figure, these mass cytokines often form an inflammatory environment, promote the proliferation and survival of LSCs, and increase their invasion and adhesion while inhibiting normal hematopoiesis. Besides, LSC-derived exosomes lead to MSC oxidative stress, affecting the exosomes secretion of MSCs and bone formation.

To make the higher efficiency in the entire leukemic endosteum microenvironment, exosomes are always utilized for distant communication between LSCs and leukemic BMM. Exosomes are small vesicles of 30–100 nm in diameter that are secreted in both normal and malignant cells, traffic mRNAs, microRNAs or small exosome proteins to affect the recipient cells or distant tissues (Kourembanas, 2015; Paggetti et al., 2015). Kourembana S thoroughly summarized the progression of exosomes production and secretion. More importantly, in multiple clinical trials and animal models, exosomes from MSCs significantly alleviate the symptoms of pulmonary hypertension, right ventricular hypertrophy, and bronchopulmonary dysplasia, it points out the immunomodulatory and anti-inflammation function of exosomes from MSCs (Kourembanas, 2015). Besides, HSCs and MSCs have physiological exosome-based communication in bone marrow, but bidirectional exosome changes vary under disease conditions as with cytokines. Particularly, the exosomes secreted by leukemia cells are largely taken up by MSCs and endothelial cells (Kumar et al., 2018); and even in the case of distant leukemia cells, MSCs are still greatly affected (Huan et al., 2015), then this process in turn affects the synthesis and secretion of MSC exosomes (Viola et al., 2016). In fact, as previously mentioned, exosomes influence LSCs and MSCs function such as cytokines secretion, proliferation (Roccaro et al., 2013), osteogenesis (Kumar et al., 2018), adhesion migration (Corrado et al., 2014; Corrado et al., 2016), chemoresistance (Viola et al., 2016) and even physiological hematopoiesis impairment (Huan et al., 2015). Specifically, leukemic exosomes initiatively deregulated normal hematopoiesis through disturbing CXCL12, SCF, KITL, IL-8, MMP9, and so on (Huan et al., 2015; Corrado et al., 2016; Kumar et al., 2018). MicroRNAs from MSC exosomes even induce DNA damage and mutagenesis of HSCs for MDS initiation (Meunier et al., 2020). Moreover, exosomes from MSCs even contain miR-155, a well-established microRNA that regulates hematopoietic malignancy (Viola et al., 2016). Strikingly, exosomes may be a major interference of communication between LSCs and MSCs, because it has been reported that exosomes from LSC sources induce nitric oxide elevation (Jafarzadeh et al., 2018), endoplasmic reticulum stress, unfolded protein response (Doron et al., 2018), and TGF-β/CXCL12/CXCR4 axis in MSCs to make an inflammatory environment; and those have been reported to change MSCs into cancer-associated fibroblasts in CLL and B-ALL (Paggetti et al., 2015; Pan et al., 2020). Therefore, exosome is another important way for LSC-MSC communication (Figure 3). Perhaps inhibiting or cutting off the formation of abnormal exosomes or creating vehicles of exosomes-associated drug delivery system can make a more effective treatment for leukemia, but there is still a long way to go.

## MSCs Support Survival and Proliferation of LSCs

Various mesenchymal stem cells seem to support leukemogenesis, even differentiated osteoblasts and adipocytes support leukemia progression (Brenner et al., 2017; Shafat et al., 2017). Changes in the BMM seem to be highly specific for oncogenic events in leukemia cells (Krause and Scadden, 2015). No matter how many changes in the subtypes of MSC, their common characteristics is secreting abundant SCF, CXCL12, VCAM1, and so on to maintain HSCs (Frenette et al., 2013); therefore, these chemokines can be the most important ones of the criteria for judging whether it is a leukemic niche-positive MSC. Leukemic MSCs undergo inflammation and can support a variety of malignant hematopoietic disorders (Ping et al., 2019). For instance, MSCs can enforce LSC survival and adhesion, in part, through the secretion of various inflammatory mediators including TNFα, CXCL1, CCL2, IL-8, and CD44 adhesion molecule (Quere et al., 2011; Agarwal et al., 2021). Pathways including cell-to-cell conjunction, extracellular matrix (ECM) molecules, extracellular matrix remodeling, and cytokine-receptor interactions are involved in MDS MSC-LSC, benefiting LSCs’ homing, harboring, and proliferation (Medyouf et al., 2014; Kouzi et al., 2020). MSCs have been reported to support AML cell survival, BM homing, and promote chemoresistance. Further, AML-induced osteogenic differentiation in MSCs supports leukemia growth (Nwajei and Konopleva, 2013; Battula et al., 2017). Moreover, the MSCs also support the long-term proliferation of the AML cells with increased phosphorylation of mTOR and its downstream targets (Brenner et al., 2017). On the other hand, evidence indicates that malignant cells actively shape their microenvironment to reinforce disease progression at the expense of hematopoiesis (Schepers et al., 2015; Hoggatt et al., 2016). The status (such as the proliferation and differentiation, self-renewal, LSC harbor, hematopoietic support) of MSCs is different in different types of leukemia, along with the change of HSC-supported CXCL12, inflammatory TNFα, NF-κB signaling, proliferated WNT-β-catenin signaling, and so on. Undoubtedly, the widespread presence of MSCs expands the scope of its hematopoietic environment support. MSCs co-localize with HSCs and LSCs in the BM niche and influence their fate decision through mutual crosstalk. The influence of MSC on the leukemogenesis could be attributed, in part, to their immune modulation behavior and tendency for tumor prone.

## MSC Changes in the Entire Bone Marrow Microenvironment as the Mediator

In fact, BMM is a complex network for HSCs and HSCs support, multiple cells and tissues are directly or indirectly linked to influence HSCs and LSCs homeostasis. For example, sympathetic nerves, which are intertwined with small arterial vessels, provide geographic location for MSCs and secrete Adrβ3 to regulate CXCL12 secretion of MSCs under physiological conditions (Agarwala and Tamplin, 2018). However, MLL-AF9 leukemic cells denervate sympathetic nerve fibers to release MSC proliferation inhibition, which enforce MSCs to proliferate into osteogenic progenitor cells through Adrβ2 (Hanoun et al., 2014). Surprisingly, JAK2 V617F mutant MPN produces excessive inflammatory IL-1β that damages both neural and CXCL12-abundant Nestin + MSCs (Arranz et al., 2014), and adrenaline is proved to be a key hormone regulating LSCs-SNS-MSCs axis.

MSCs are widely considered to be immunosuppressors (Maccario et al., 2005; Shi et al., 2010). All MSCs, which are widely present in the body, can undergo immune and inflammatory regulation. It is generally believed that MSC rapidly responds to homeostasis in the body. Excessive IFN and the interleukin family induce the secretion of IFN, IDO, and iNOS in MSCs, thereby inhibit the function of T cells, NK cells, and DCs maturation. Meanwhile, it promotes immunosuppressive Tregs proliferation (Singer and Caplan, 2011; Vasold et al., 2015). In addition, many accessory cells, such as T cells, B cells, and DCs are also involved in the hematopoiesis (Frenette et al., 2013). The dysfunction of T cells and the proliferation of Tregs are well-known in high-risk MDS and CML patients, and they are strongly associated with changes in MSCs (Ganan-Gomez et al., 2015; Brück et al., 2018). For example, under physiological conditions, MSCs are thought to inhibit the maturation of DC cells (Jiang et al., 2005), but Zhao ZG et al. found that CML MSCs can activate regulatory DCs, thereby inhibiting T cell function or promoting Tregs proliferation, and indirectly participating in immune escape (Zhao et al., 2012). Recently, it has been reported that inflammatory factors TNF-α and TFN-γ can promote MSCs to produce a large amount of PD-L1 and PD-L2, which bind to the PL-1 of T cells and inhibit the activation of T cells to promote immune escape (Davies et al., 2017), but it has not yet begun to use the immune checkpoint blockade in leukemia. However, in fact, there is no deep understanding of the specific immunosuppressive mechanism of MSCs in leukemia, thus, more evidence is needed.

## Therapeutic Targeting of MSCs in Myeloid Leukemia

MSCs have strong hematopoietic support ability, wide sources, and low immunogenicity. So, the first participation of MSC in leukemic clinical trials is bone marrow transplantation. MSCs are always used to infuse with HSC for better hematopoietic recovery, avoiding and ameliorating graft versus host disease (Zhao and Liu, 2016). MSCs can promote HSC colonization and hematopoietic homeostasis. However, since LSCs and HSCs share the same bone marrow microenvironment, minimal resident LSCs are more likely to reprogram donor MSCs for its expansion and leukemia relapse (Jin et al., 2008; Agarwal et al., 2019).

On the other hand, LSCs build its microenvironment through both physical adhesion and cytokine-receptor interaction. Hence most of therapeutic targets of MSCs are used to exert or enhance the efficacy of chemotherapy drugs. In general, although numerous medicines are still under research, there are currently four main medicine types that entering clinical trials, namely chemo-sensitizing medicines, chemotherapy synergistic medicines, adhesion inhibitors, and bone homeostasis medicines.

Among them, CXCL12/CXCR4 inhibitors as the first chemo-sensitizing drugs are the earliest ones that entered clinical trials (Ladikou et al., 2020). Since LSCs still need MSC-derived CXCL12 to maintain its self-renewal and chemotherapy resistance (Agarwal et al., 2019), blocking CXCL12/CXCR4 axis can inhibit the protective effect of MSC on LSCs, and increase the sensitivity of chemotherapy to LSC. Lots of CXCR4 antagonist, such as plerixafor (Uy et al., 2012; Borthakur et al., 2020), LY2510924 (Boddu et al., 2018), BL-8040 (Borthakur et al., 2021), POL6326 (Chen et al., 2010), etc. have been applied in clinical trials in AML patients recently. At the same time, plerixafor also can synergize with chemotherapeutic drugs to mobilize LSC for myeloablation and subsequent allografting (Michelis et al., 2019).

Besides, chemotherapy synergistic drugs act by synergistically inhibiting the function of LSCs and MSCs, such as inhibitors of the WNT/β-catenin signaling pathway (Zhou et al., 2017; Jiang et al., 2018). Canonical WNT/β-catenin signaling pathway is considered to be critical for the stemness of LSCs and MSCs (Stoddart et al., 2016; Carter et al., 2019). These medicines simultaneously target myeloid leukemia cells and MSCs to exert a synergistic killing effect. At present, a variety of inhibitors of the WNT/β-catenin signaling pathway also have entered clinical trials, such as CWP232291 (Lee et al., 2020).

Since MSCs physically contact LSCs for its protection, inhibiting the adhesion of LSCs to MSCs is also an effective method to prevent LSC homing and increase chemosensitivity. At present, the main adhesive targets are CD44 (Gutjahr et al., 2021; Yu et al., 2021), E-selectin (DeAngelo et al., 2018) and CLA-4 (Nair-Gupta et al., 2020; Gutjahr et al., 2021), which have been confirmed to protect LSCs through direct adhesion of MSC-LSC. The anti-CD44 antibody (ARH460-16-2) and the E-selectin antagonist (Uproleselan) have entered Phase I/II clinical trials (Vey et al., 2016; DeAngelo et al., 2018), but the study of VLA-4 is also in progress.

Finally, the bone homeostasis medicines that are designed to enforce MSC osteo-differentiation remodel leukemia BMM and induce apoptosis of leukemia cells. Those medicines expel LSCs from its MSC-enriched microenvironment and suffer environmental stress. Proteasome drugs are mainly used in leukemia for bone remodeling. For now, Carfilzomib (Berdeja et al., 2015) and Ixazomib (Advani et al., 2019) have entered phase I/II clinical trial. They can promote osteoblast differentiation of MSCs and induce apoptosis of leukemia cells.

Overall, the therapeutic targets of MSCs are divided into chemo-sensitizing and broad-spectrum chemotherapeutics for both MSCs and LSCs, and lots of treatment strategies are in clinical trials. But it is worth noting that although leukemia cells use BMM molecules for its benefits, those molecules are also regulators of physiological HSC self-renewal and mobilization. Therefore, thoroughly removing the protection of MSCs to LSCs while minimizing its impact on physiological HSCs will be the best therapeutic solution.

## Discussion

In this article, we mainly explored the role of MSCs in malignant myeloid leukemia and explained the systemic changes of MSCs in myeloid leukemia from various aspects. We summarized that regardless of myeloid leukemia subtype, MSCs sustain malignant hematopoietic support to LSCs. This support is widely reinforced in the LSC maintenance and protection of LSCs from stress, including the establishment of the leukemic BMM niche, residual LSCs harbor, and relative quiescent long-term LSCs maintenance. However, MSCs may not be necessary during the expansion of LSCs, which mainly depends on strong LSCs malignant proliferation ability. Therefore, combining the inhibition of LSCs expansion and the dissolution of the MSCs-LSCs niche is an effective treatment for myeloid leukemia. We also briefly discussed that the exosomes as a new cell-to-cell communication method in LSCs-MSCs niche interaction with leukemia development. Besides, the normal hematopoietic and leukemic bone marrow microenvironments are highly complex entities. Not only mutations in HSCs have been found in leukemia patients, mutations in mere MSCs also can completely induce leukemia in mice models, which has greatly improved the role of MSCs in leukemogenesis. However, the current research on the role of MSCs in leukemia is still very shallow with many unsolved mysteries, and it will take a long time for clinical application. Clearly, MSCs should be viewed as a double-edged weapon, hence, further research is recommended to thoroughly understand the complex interactions between LSCs and the surrounding microenvironment.

Recent and current studies have highlighted the niche role in leukemia progression, but the detailed mechanism is still unknown. Besides, how the BMM influences treatment results or if it contains any potential target for treatment is needed to be explored. Further studies need to address the following directions: 1. The easiest place to apply MSCs is the diagnostic grade and prognosis of leukemia. Kim et al. suggested that changes in microenvironment especially MSCs can be used as a criterion for diagnosis, treatment, and prognosis of AML (Kim et al., 2015; Kornblau et al., 2018); however, what specific markers can be used as a diagnosis basis for clinic is still challenged. Because of the ability of MSCs for leukemogenesis, patients with abnormal myeloid hyperplasia also need to detect chromosomal mutations in MSCs. 2. At present, it only reveals the direct influential reaction of MSCs and LSCs interaction, and only stays at the surface stage without deep exploration. More in-depth molecular mechanisms, immune suppression, immune evasion, and the mechanism of chemo-resistant LSCs harbor, as well as the detailed mode of action of exosomes, remain largely unknown and still require significant efforts. When these problems are correctly recognized, the true pathological mechanism of leukemia and leukemic MSCs can be understood, and a truly effective solution can be made. 3. The most important thing is that it can be used for the treatment of leukemia. At present, it is difficult to completely cure leukemia with single leukemia treatment measure due to the frequent chemo-resistance and recurrence. Because of the great difference of leukemic MSCs compared to normal MSCs, it is obvious that the targeted leukemic MSCs synergistic chemotherapy drug can be used more effectively and prevents drug resistance and recurrence. For example, it has been reported that Pml is not only essential for LSCs, but also important for MSCs to maintain leukemia process (Guarnerio et al., 2018). Decreasing the expression of Pml in MSCs could inhibit LSCs proliferation, and this may become an effect therapy (Guarnerio et al., 2018). In addition, increased knowledge on how LSCs develop in their BM niche might be therapeutically exploited to block leukemia progression and target LSCs while sparing normal HSCs. In summary, although the outlook of leukemic MSCs is considerate, in-depth study of MSCs and associated the normal hematopoietic and leukemic bone marrow microenvironments in leukemia still has a huge developmental potential.
